# Comparison of porcine thorax to gelatine blocks for wound ballistics studies

**DOI:** 10.1007/s00414-015-1309-9

**Published:** 2016-02-04

**Authors:** A. Mabbott, D. J. Carr, S. Champion, C. Malbon

**Affiliations:** 1 Impact and Armour Group, Centre for Defence Engineering, Cranfield University at the Defence Academy of the United Kingdom, Shrivenham, SN6 8LA UK; 2 Weapons and Vehicle Systems Group, Centre for Defence Engineering, Cranfield University at the Defence Academy of the United Kingdom, Shrivenham, SN6 8LA UK; 3 Mechanical Engineering, Material Science and Civil Engineering, Centre for Applied Science and Technology, Home Office Science, Woodcock Hill, Sandridge, St Albans, Hertfordshire AL4 9HQ UK

**Keywords:** Tissue simulants, Pistol, Rifle, 10 and 20 % gelatine

## Abstract

Tissue simulants are typically used in ballistic testing as substitutes for biological tissues. Many simulants have been used, with gelatine amongst the most common. While two concentrations of gelatine (10 and 20 %) have been used extensively, no agreed standard exists for the preparation of either. Comparison of ballistic damage produced in both concentrations is lacking. The damage produced in gelatine is also questioned, with regards to what it would mean for specific areas of living tissue. The aim of the work discussed in this paper was to consider how damage caused by selected pistol and rifle ammunition varied in different simulants. Damage to gelatine blocks 10 and 20 % in concentration were tested with 9 mm Luger (9 × 19 full metal jacket; FMJ) rounds, while damage produced by .223 Remington (5.56 × 45 Federal Premium® Tactical® Bonded®) rounds to porcine thorax sections (skin, underlying tissue, ribs, lungs, ribs, underlying tissue, skin; backed by a block of 10 % gelatine) were compared to 10 and 20 % gelatine blocks. Results from the .223 Remington rifle round, which is one that typically expands on impact, revealed depths of penetration in the thorax arrangement were significantly different to 20 % gelatine, but not 10 % gelatine. The level of damage produced in the simulated thoraxes was smaller in scale to that witnessed in both gelatine concentrations, though greater debris was produced in the thoraxes.

**Electronic supplementary material:**

The online version of this article (doi:10.1007/s00414-015-1309-9) contains supplementary material, which is available to authorized users.

## Introduction

Many tissue simulants have been and continue to be used in the study of ballistics as substitutes for biological tissues such as skin, muscles and organs, e.g. [1, 2]. Perhaps the most widely used simulants are gelatine and glycerine soap [3]. Gelatine is typically utilised in either a 10 %, e.g. [1, 2, 4–8] or 20 %, e.g. [9–11] (by mass) concentration, conditioned at 4 and 10 °C, respectively. Early studies found that using gelatine produced similar penetration depths for a range of ammunition to those observed in soft tissue, while demonstrating the mechanics of the temporary and permanent cavities that resulted from a ballistic impact [9, 12, 13]. Gelatine is translucent in nature meaning a projectiles’ behaviour and the exact path and placement of projectiles and/or fragments can be easily viewed and analysed [1, 7, 14, 15]. However, no agreement as to which gelatine concentration to utilise has been reached, nor a standard method for preparation [2, 16]. Work in the open source literature which uses and compares how both gelatine concentrations fare in ballistic testing is limited, e.g. [11, 12, 17].

In a gunshot wound in living tissue, three zones are used to describe the areas of the wound, the central zone, caused by the direct crushing and lacerating of tissue by the projectile, surrounded by the second and third zones, contusion and concussion, respectively [18–20]. The outer two zones are believed to be the result of the temporary cavitation process, with the zone of contusion consisting of non-viable tissue, and the concussion zone showing damaged tissue capable of recovering [21]. Variables including shape, size, likelihood of fragmentation, mass, velocity and available kinetic energy of the projectile, together with the variable physical characteristics of the living target, all have an effect on the damage that is produced [20, 22–24].

Questions still remain regarding how damage recorded in tissue simulants compares to damage in living tissue and to specific areas of a human body. Although gelatine has been shown to be a close match for thigh muscle of both humans and pigs when comparing densities [2, 21, 25, 26], as well as 10 % gelatine being shown to produce depths of penetration that are within 3 % of living porcine muscle [4, 5], a typical priority area on a human target is not the thigh muscle. An area of the body that is more commonly targeted during a ballistic attack is the thorax, which is composed of many differing materials (skin, muscle, bone, heart, lungs, blood vessels, fatty deposits, nerves, etc.) and is thus very different to the composition of thigh muscle.

Breeze et al. [27] found significantly different depths of penetrations were produced in the thorax and abdomen of pig cadavers compared to 20 % gelatine, when testing three different fragment simulating projectiles (FSPs). The outcome was attributed to the anatomical complexity and multiple tissue interfaces of the thorax and abdominal regions.

The aim of the work discussed in this paper was to consider how damage to a tissue simulant compares to damage observed in a thorax after ballistic attack. Following previous work [28, 29], porcine thoracic walls were utilised either side of a pair of lungs to simulate a thorax, with results being compared to 10 and 20 % gelatine blocks.

## Materials and methods

### Materials

Gelatine from a single manufacturing batch and with a Bloom strength of 225–265 (type 3 ballistic photographic grade gelatine^1^) was used to manufacture 10 and 20 % gelatine blocks. The moulds in which the gelatine blocks were manufactured measured 250 mm (w) × 250 mm (h) × 500 mm (l), with both longer sides tapered 1° to facilitate set gelatine block removal. Both gelatine concentrations were left to set at room temperature (~18 °C ± 3 °C) for 24 h, before being placed in a refrigerator for a further 24 h; 10 % blocks at 4 °C, 20 % blocks at 10 °C prior to use.

Samples of porcine thoracic walls^2^ (consisting of the ribs, intercostal muscles, underlying tissue and skin; vertebra and the sternum removed) and sets of porcine lung pairs complete with trachea were collected and kept refrigerated one day prior to testing. Samples were brought up to room temperature for at least 12 h before testing (~18 ± 3 °C). The samples used were all of food-grade standard and fit for human consumption; consequently, there were no ethical concerns raised for this study

**Fig. 1:**
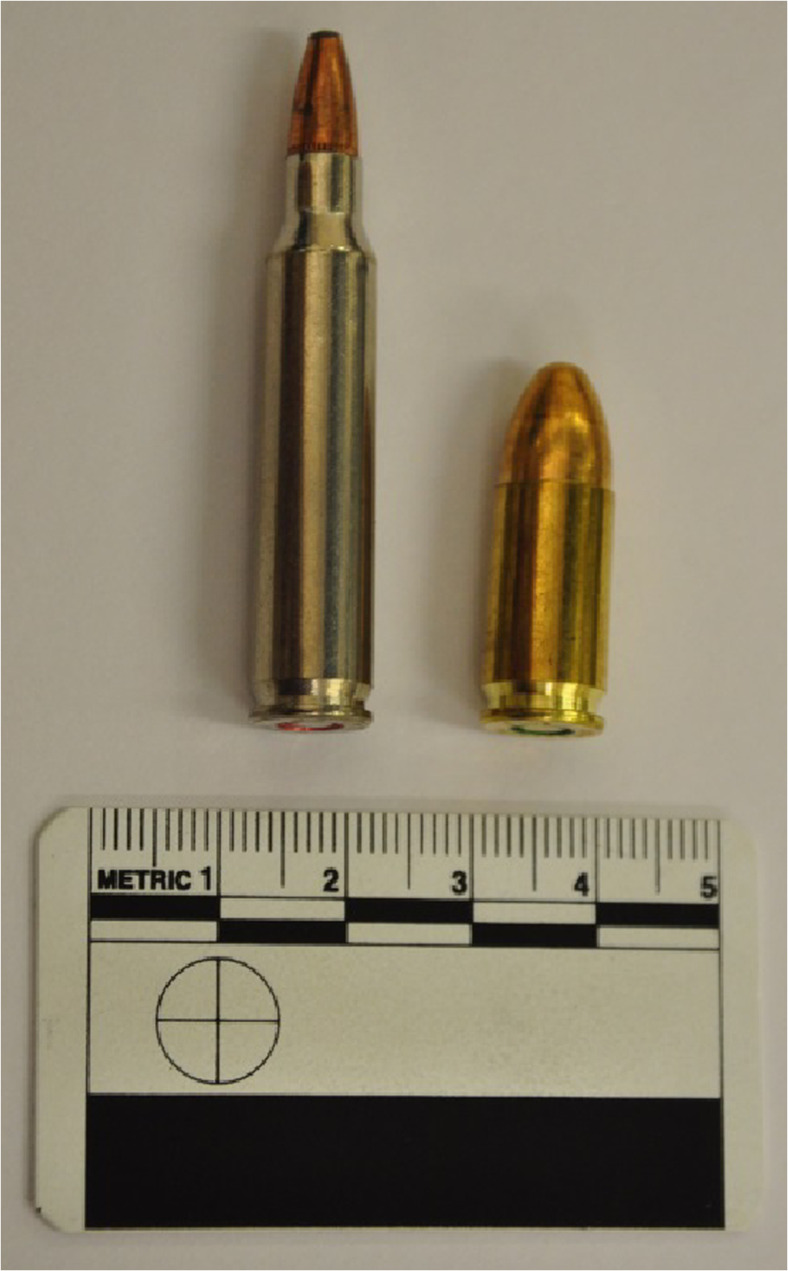
.223 Remington (5.56 × 45; 62 grain; Federal Premium® Tactical® Bonded®) (*left*) and 9 mm Luger (9 × 19 FMJ; 124 grain; DM11 A1B2)* (right)*

Two ammunition types were used for testing:

(i) .223 Remington (5.56 × 45; 62 grain; Federal Premium® Tactical® Bonded®)
(ii) 9 mm Luger (9 × 19 FMJ; 124 grain; DM11 A1B2) (Fig. 1)

The two types of ammunition cover both a rifle and a pistol round, both rounds are designed to interact differently with targets. The .223 Remington rifle round has an exposed tip and typically expands on impact, while the 9 mm Luger pistol round does not typically breakup or fragment, but does have a tendency to yaw within targets.

### Methods—gelatine testing

All testing was performed at the Small Arms Experimental Range at Cranfield University. The targets were placed 10 m down range from the end of the muzzle. An Enfield Number 3 Proof Housing, with the appropriate barrel fitted, was used to fire each ammunition type. Ten shots with each ammunition type was carried out (*n* = 10). A new gelatine block was used for every shot with the .223 Remington ammunition, while two or three rounds of the 9 mm Luger ammunition were fired into each gelatine block, ensuring the tracts did not overlay. The impact velocities were recorded using a Weibel W-700 Doppler radar, and the impact event was recorded using a Phantom V12 high-speed video camera (41,025 fps, 5 μs exposure time and 512 × 256 frame resolution).

Prior to testing, a 5.5-mm-diameter steel BB was fired at ~750 m/s from a distance of 10 m into the top right of each gelatine block. The velocity and depth of penetration of these shots were measured and compared with results collected from previously published depth of penetration testing to ensure only calibrated gelatine blocks were used for testing [17].

### Methods—porcine thorax testing

**Fig. 2:**
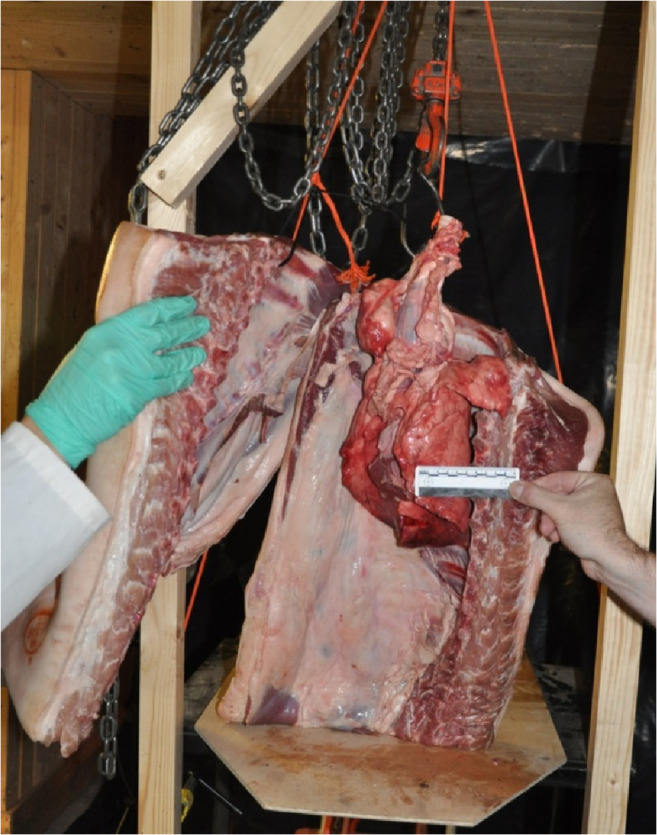
Typical set up showing the arrangement of the thoracic walls and lungs

The porcine samples were arranged 10 m down range from the end of the muzzle to simulate a thorax; a porcine thoracic wall was placed as the anterior of the target (skin facing muzzle), then a set of lungs positioned in relation to the thoracic wall as they would be anatomically in a human, followed by another thoracic wall (skin facing away from muzzle) (Fig. 2). In order to ensure that a complete bullet tract was captured, a 10 % gelatine block 500 mm in length was placed adjacent to and in contact with the posterior thoracic wall. An Enfield Number 3 Proof Housing, with the appropriate barrel fitted, was used to fire each ammunition type. Each individual shot was aimed with the goal of striking: a rib within the anterior thoracic wall, either the left or right lung, and a rib in the posterior thoracic wall, before capturing the rest of the tract in a gelatine block (calibrated as above). Shots that were fired onto the same thoracic sections were located to ensure damaged areas did not overlap. The impact velocities were recorded using a Weibel W-700 Doppler radar, and the impact event was recorded using a Phantom V12 high-speed video camera (21,005 fps, 5 μs exposure time and 512 × 512 frame resolution). Ten shots in total were carried out (*n* = 7 for .223 Remington; *n* = 3 for 9 mm Luger).

### Analysis

#### Gelatine blocks

**Fig. 3:**
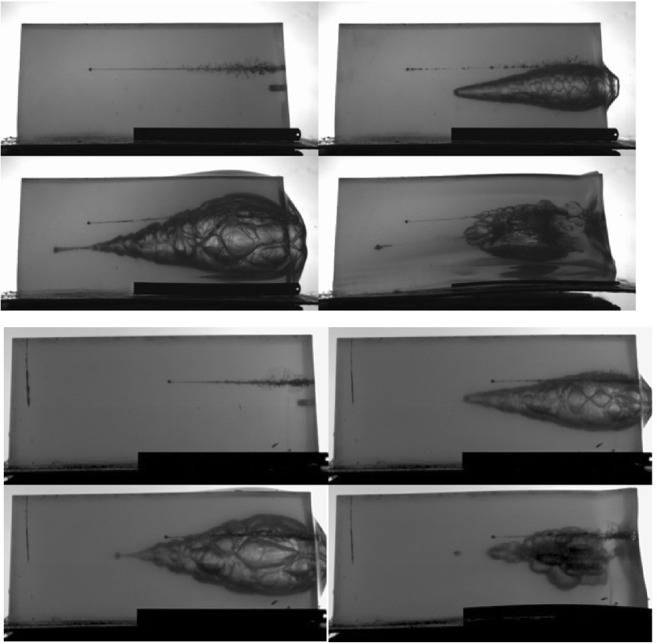
High-speed stills of a typical .223 Remington impact into 10 % gelatine (*top four images*) and 20 % gelatine (*bottom four images*)

Analysis of the high-speed footage was carried out using Phantom software (Vision Research, Phantom Camera Control Application 2.5) (Fig. 3). Each file was calibrated by using a known length visible in the image, converting pixels present in the image to a dimension in millimeters. Once calibrated, it was possible to take measurements that included the diameter of the temporary cavity at its largest, and the distance (from the entry point of the projectile in the gelatine block) to where this occurred. Both these measurements were taken using the PCC 2.5 software. It was also possible to locate the position and the number of times the 9 mm Luger rounds yawed within the target.

The gelatine blocks were dissected after testing by cutting along the permanent tract using a knife. Lead debris present in the cavities was noted, photographed,^3^ removed and bagged. Measurements of the permanent cavities (as indicated by damaged area / fissures) produced in the gelatine blocks were taken, specifically neck length, ‘body’ length, ‘body’ width, ‘body’ height and (when possible) distance to projectile. From the body dimensions, the formula for calculating the volume of an ellipsoid was used to calculate a representation of the maximal volume of the damage the permanent cavity created:

3.1 $$ {V}_{\mathrm{ellipsoid}}\kern0.5em =\kern0.5em \frac{4}{3}\ \pi\ lwh, $$

where *l*, *h* and *w* are the length, width and height body dimensions, all halved. Length and height of fissures present in the gelatine blocks were also recorded. From these measurements, the area of each individual fissure was calculated using the formula for an ellipse, divided by two as a fissure was only half of an ellipse:

3.2 $$ {A}_{\mathrm{ellipse}}\kern0.5em =\kern0.5em \frac{\pi ab}{2}, $$

where *a* and *b* are the respective length and height measurements of a fissure, halved. A total fissure area for each shot was calculated by adding together the areas of the individual fissures.

Summary statistics (mean (x), standard deviation (s.d.) and coefficient of variation (CV)) were calculated for the fissure and the permanent cavity data sets, as well as the data from the high-speed video analysis. Analysis of variance (ANOVA) was used to determine when significant differences between the two gelatine concentrations occurred (SPSS Statistics 22.0). Normality of data and equality of variance were checked for each data set.

#### Porcine tissue

Post firing analysis of the porcine thoracic walls and lungs was performed after all shots had been completed. Measurements of the entrance and exit wounds of each shot were taken from every perforated section of each simulated thoracic cavity (i.e. the front thoracic wall, the lung and the rear thoracic wall). Any projectile and/or bone fragments found were photographed, weighed^4^ and recovered, before dissection of the samples took place. Further fragments found from exploration of the damage were also photographed, recovered and weighed.

Post firing analysis of the gelatine blocks located behind the porcine tissue consisted of cutting along the length of the permanent cavity, before measurements of the cavity were taken. When present, the projectile and any projectile and/or bone fragments were photographed, recovered and weighed.

ANOVA and Tukey analysis were used to determine if there were significant differences amongst the distance to projectile data obtained from firing .223 Remington projectiles at the porcine thoracic target arrangement, 10 % gelatine targets and 20 % gelatine targets (SPSS Statistics 22.0). Normality of data and equality of variance were checked for each data set.

## Results

### .223 Remington projectiles

Comparing the two gelatine concentrations revealed that the mean measurements of both the temporary and permanent cavities in 10 % gelatine were larger than those collected from cavities in 20 % gelatine blocks (Table 1). The spread of the data was also typically larger for the measurements collected from 10 % gelatine (Table 1). Metallic deposits were found within all targets, none greater than 0.5 mm in size.

**Table 1:** Results collected from interactions between .223 Remington and (a) 10 and (b) 20 % gelatine

(a) 10 % gelatine									
Shot no.	Impact velocity (m/s)	Temporary cavity		Permanent cavity					Distance to projectile (mm)
		Distance to maximum expansion (mm)	Maximum diameter (mm)	Neck length (mm)	Body length (mm)	Body height (mm)	Body width (mm)	Total fissure area (mm^2^)	
1	843	85	178	0	300	95	105	47,000	454
2	844	91	180	0	280	100	110	40,000	425
3	842	75	177	2	260	100	115	45,200	425
4	852	77	171	0	325	90	105	76,000	423
5	853	100	174	0	330	105	105	81,300	423
6	852	99	182	0	340	145	100	59,300	420
7	853	102	179	0	290	140	95	85,000	402
8	839	85	178	0	320	110	150	77,500	430
9	844	93	184	0	315	110	130	67,400	428
10	854	75	173	0	325	125	130	111,000	429
Mean	847.6	88.7	178.0	N/A	308.5	112.0	114.5	69,000	425.9
s.d.	5.7	10.3	4.0	N/A	25.3	18.7	17.1	22,000	12.7
CV (%)	0.7	11.7	2.2	N/A	8.2	16.7	14.9	31.7	3.0
Min	839	75	171	0	260	90	95	40,000	402
Max	854	102	184	2	340	145	150	111,000	454
(b) 20 % results									
Shot no.	Impact velocity (m/s)	Temporary cavity		Permanent cavity					Distance to projectile (mm)
		Distance to maximum expansion (mm)	Maximum diameter (mm)	Neck length (mm)	Body length (mm)	Body height (mm)	Body width (mm)	Total fissure area (mm^2^)	
1	839	88	161	5	240	95	85	58,000	315
2	842	88	158	1	260	100	85	42,000	316
3	842	86	163	1	270	115	110	36,900	306
4	844	83	153	0	260	110	110	53,300	295
5	845	83	163	1	275	115	100	49,100	287
6	844	88	158	3	245	130	120	56,600	280
7	852	80	155	1	260	110	120	37,600	289
8	846	80	154	2	230	85	110	50,000	283
9	855	90	155	0	260	115	95	57,000	299
10	841	84	156	0	245	95	110	62,000	292
Mean	845.0	85.0	157.6	1.4	254.5	107.0	104.5	50,000	296.2
s.d.	5.0	3.5	3.6	1.6	14.0	13.2	12.8	8,800	12.7
CV (%)	0.6	4.1	2.3	112.7	5.5	12.3	12.2	17.4	4.3
Min	839	80	153	0	230	85	85	36,900	280
Max	855	90	163	5	275	130	120	62,000	316

Analysis of variance on the temporary cavity measurements revealed that the distance to the maximum expansion in both concentrations of gelatine was not significantly different (*F*_1, 18_ = 1.12, *p* = NS), though the mean distance was numerically greater in 10 % gelatine (Table 1). Conversely, the maximum expansion (diameter) reached by the temporary cavity was significantly affected by gelatine concentration (*F*_1, 18_ = 144.25, *p* ≤ 0.001). The mean temporary cavity was larger in 10 % gelatine (mean = 178.1 mm, s.d. = 4.0 mm), when compared to the mean temporary cavity diameter in 20 % gelatine (mean = 157.6 mm, s.d. = 3.6 mm).

From the permanent cavity data collected, analysis of variance could not be carried out on neck length due to there being only one measurement in 10 % gelatine. For body length, however, gelatine concentration had a significant effect (*F*_1, 18_ = 34.88, *p* ≤ 0.001). The mean body length was longer in 10 % gelatine (mean = 308.5 mm, s.d. = 25.3 mm) compared to 20 % gelatine (mean = 254.5 mm, s.d. = 14.0 mm). The representation of the maximum ellipsoid volume was significantly different in both concentrations of gelatine (*F*_1, 18_ = 9.08, *p* ≤ 0.01). The mean volume was larger in 10 % compared to 20 % gelatine (10 % gelatine mean = 2,100,000 mm^3^, s.d. = 50,000 mm^3^; 20 % gelatine mean = 1,500,000 mm^3^, s.d. = 330,000 mm^3^). Concentration of gelatine also significantly affected fissure area of the permanent cavity (*F*_1, 18_ = 6.36, *p* ≤ 0.05). The mean area in 20 % gelatine (mean = 50,000 mm^2^, s.d. = 8700 mm^2^) was less than the mean fissure area in 10 % gelatine (mean = 69,000 mm^2^, s.d. = 22,000 mm^2^).

The distance .223 Remington projectiles penetrated within the different gelatine concentrations was significantly different (*F*_1, 18_ = 524.51, *p* ≤ 0.001). The mean distance in 10 % gelatine (mean = 425.9 mm, s.d. = 12.7 mm) was 129.7 mm longer than the mean distance to projectile in 20 % gelatine (mean = 296.2 mm, s.d. = 12.7 mm).

### .223 Remington simulated thorax testing

Seven shots were carried out into the simulated thorax targets (Table 2). Tissue and metallic debris from all porcine samples was collected and weighed (see electronic supplementary material).

**Table 2:** Results collected from interactions between .223 Remington projectiles and simulated thoraxes

Shot no.	Impact velocity (m/s)	Entry location			Distance through thoracic samples (mm)	Distance in 10 % gelatine (mm)	Total distance (mm)
		Anterior thoracic walls	Lungs	Posterior thoracic walls			
1	852	Nicked rib 5	Right lung, nicked the top of the inferior lobe	Hit rib 3	177	299	476
2	851	Hit rib 5	Left lung, top area of the inferior lobe	Hit rib 4	177	245	422
3	847	Hit rib 7	Left lung, middle area of the superior lobe	Hit rib 7	165	295	460
4	845	Hit rib 5	Right lung, nicked the top edge of the middle lobe	Between ribs 3 and 4	165	332	497
5	840	Hit ribs 5 and 6	Right lung, top area of the inferior lobe	Hit rib 5	170	287	457
6	837	Hit rib 7	Left lung, bottom area of the superior lobe	Hit rib 6	170	269	439
7	847	Hit rib 8	Left lung, middle area of the inferior lobe	Hit rib 8	170	299	469

In order to compare the distances to which .223 Remington projectiles penetrated the simulated thoraxes with the distances produced in both gelatine concentrations, only the first seven shots into the respective gelatine blocks were used for ANOVA, ensuring equality of sample size. Target material had a significant effect on the distance to the projectiles travelled (*F*_2, 18_ = 146.54, *p* ≤ 0.001). Tukey’s HSD multiple comparison test indicated the three different target types resulted in three varying levels of distances travelled. Distance was greatest in the simulated thorax cavity arrangement (mean = 460.0 mm, s.d. = 24.5 mm); mean distance in 10 % gelatine was slightly shorter (mean = 424.6 mm, s.d. = 15.2). Mean distance in 20 % gelatine was over 160 mm shorter compared to the thoracic cavity (mean = 298.3 mm, s.d. = 14.2 mm) (Tables 1 and 2). Comparison of the respective CVs revealed that the variability of the thoracic cavity was similar to those produced in the gelatine targets.

Studying the strike location through the thoracic cavity targets revealed that shot 4, which resulted in the longest distance to the projectile, did not fully strike a lung (caught the top edge of the right middle lobe), while also exiting the posterior thoracic wall without hitting a rib (between ribs 3 and 4). Shot 1 also only nicked the top of a lung lobe (top of the right inferior lobe), while not hitting a rib squarely when entering the anterior thoracic wall (nicked rib 5). Therefore, a further ANOVA was run with these two shots removed. The remaining five shots were compared to the first five shots from the 10 and 20 % gelatine testing. For this data sub-set, mean distance to .223 Remington projectile was significantly affected by the target material (*F*_2, 12_ = 135.09, *p* ≤ 0.001). Tukey’s HSD multiple comparison revealed that two differing levels of distances travelled by the projectiles existed; projectiles which struck the simulated thorax and 10 % gelatine blocks in one level, and shots into 20 % gelatine blocks in the other. The longest mean distances were in the simulated thoraxes (mean = 449.4 mm, s.d. = 18.8 mm), ~ 19 mm greater in length than shots into 10 % gelatine (mean = 430.0 mm, s.d. = 13.5 mm). Mean distance in 20 % gelatine blocks was a further 126.2 mm shorter (mean = 303.8 mm; s.d. = 12.6 mm).

### 9 mm Luger projectiles

The 9 mm Luger rounds perforated the gelatine blocks, regardless of concentration. The tract left by the rounds was helical in shape; there was not a ‘body’ of damage left. As a result, the permanent cavity damage was only assessed by measuring the fissure area that was present. The results revealed that fissure area measurements were typically greater in 10 % gelatine, with the range also larger in 10 % gelatine blocks (Table 3). No debris was found in any gelatine targets.

**Table 3:** Results collected from interactions between 9 mm Luger and (a) 10 and (b) 20 % gelatine

(a) 10 % gelatine								
Shot no.	Impact velocity (m/s)	Temporary cavity		Yaw				Total fissure area (mm^2^)
		Distance to maximum expansion (mm)	Maximum diameter (mm)	Number of times 90° reached	Location within block (mm)			
1	422	276	113	3	243	301	466	41,000
2	429	314	80	2	318	367	–	37,000
3	429	277	118	2	265	485	–	53,000
4	429	247	116	3	266	311	475	60,000
5	431	225	117	2	215	402	–	45,000
6	433	212	100	3	216	449	474	42,000
7	435	252	125	2	241	476	–	54,000
8	427	235	106	3	223	275	448	59,000
9	425	281	120	2	298	348	–	53,000
10	432	226	104	3	231	277	451	47,000
Mean	429.2	254.6	109.9	2.5	251.4	369.1	462.7	49,000
s.d.	3.9	31.9	13.1	0.5	35.0	80.4	12.8	8,000
CV (%)	0.9	12.5	11.9	21.1	13.9	21.8	2.7	16.2
Min	422	212	80	2	215	275	448	37,000
Max	435	314	125	3	318	485	475	60,000
(b) 20 % gelatine								
Shot no.	Impact velocity (m/s)	Temporary cavity		Yaw				Total fissure area (mm^2^)
		Distance to maximum expansion (mm)	Maximum diameter (mm)	Number of times 90° reached	Location within block (mm)			
1	420	318	71	3	285	347	476	34,000
2	434	317	85	3	247	297	447	30,000
3	427	219	76	2	209	440	–	29,000
4	427	217	99	3	201	254	406	39,000
5	432	220	87	3	222	290	446	38,000
6	427	238	93	2	225	424	–	37,000
7	420	249	79	3	230	292	409	36,000
8	422	262	86	3	234	284	426	31,000
9	420	236	99	2	226	418	–	32,000
10	427	206	62	3	235	281	417	25,000
Mean	425.6	248.1	83.6	2.7	231.4	332.7	432.4	33,000
s.d.	5.0	39.9	12.1	0.5	22.9	69.5	25.3	4,500
CV (%)	1.2	16.1	14.4	17.9	9.9	20.9	5.9	13.6
Min	420	206	62	2	201	254	406	25,000
Max	434	318	99	3	285	440	476	39,000

ANOVA identified that distance to the maximum expansion of the temporary cavity was not significantly affected by gelatine concentration (*F*_1, 18_ = 0.16, *p* = NS). The mean distance to maximum expansion was shorter in 20 % gelatine blocks (mean = 248.1 mm, s.d. = 39.9 mm), although larger variability was also witnessed in the 20 % gelatine blocks. The size of the maximum diameter of the temporary cavity was significantly affected by gelatine concentration (*F*_1, 18_ = 21.94, *p* ≤ 0.001). Mean maximum diameter was smaller in blocks 20 % in concentration (mean = 83.6 mm, s.d. = 12.0); temporary cavity size was over 35 mm larger in 10 % blocks (mean = 110.0 mm, s.d. = 13.1 mm). Variability was greater in 20 % gelatine blocks.

The mean distance to where 9 mm Luger yawed to 90°, for the first and second time respectively, was not significantly affected by gelatine concentration (*F*_1, 18_ = 2.29, *p* = NS; *F*_1, 18_ = 1.17, *p* = NS). Not all shots yawed three times; five shots did in 10 % gelatine, and seven shots in 20 % gelatine. Using this sub-set of data, gelatine concentration significantly affected the mean location of where yaw for a third time occurred (*F*_1, 10_ = 0.02, *p* ≤ 0.05). The mean distance to third yaw was longer in 10 % gelatine (mean = 462.7 mm, s.d. = 12.8 mm) when compared to 20 % gelatine (mean = 432.4 mm, s.d. = 25.3 mm).

ANOVA of the permanent cavity revealed that fissure area was significantly different (*F*_1, 18_ = 30.15, *p* ≤ 0.001). Mean area was less in 20 % than in 10 % gelatine (20 % gelatine mean = 33,000 mm^2^, s.d. = 4500 mm^2^; 10 % gelatine, mean = 49,000 mm^2^, s.d. = 7900 mm^2^).

### 9 mm Luger simulated thorax testing

All 9 mm Luger shots perforated both the simulated thoracic cavity and the 500 mm gelatine block at the rear of the target. As a result, no analysis into the distance to the projectiles was carried out. Raw data collected from the interactions with the simulated thoracic cavities are presented in the electronic supplementary material.

## Discussion

### Gelatine

The expansion of the .223 Remington rounds in both concentrations of gelatine produced temporary cavities that expanded beyond the diameter of the projectile on initial penetration, with no initial channel present beforehand (Fig. 3). The formation of the temporary cavities in both concentrations of gelatine followed the same pattern, supported by the result that there was no significant difference in the distance to the maximum point of temporary cavitation. Every shot was captured completely in the block (for both concentration types). The permanent cavity left in both concentrations of gelatine was reminiscent of an ellipsoid. Both the permanent and temporary cavities produced by .223 Remington projectiles were similar in shape and formation in both gelatine concentrations. However, greater damage was observed in 10 % gelatine blocks; with both significantly larger temporary cavity diameters and significantly larger permanent cavity measurements recorded when compared to 20 %. Although 20 % gelatine has a higher density (1.05 g/cm^3^ compared to 1.03 g/cm^3^) [25, 26], and materials of greater density absorb more energy and thus have a higher potential for sustaining damage [23], the elasticity and gel strength also affects the level of damage. Blocks of 20 % gelatine contained a higher concentration of gelatine and thus a greater gel strength [30, 31]. The greater gel strength meant the blocks were better at resisting the disruptive effects of the temporary cavity. As a result, blocks of 10 % gelatine were less efficient at containing the expansion of the temporary cavity, with less gel strength also having an effect on recovery, explaining why greater permanent damage was also produced in 10 % blocks. When measurements of fissures were compared, a similar result was seen; the areas of the fissures were larger in 10 % blocks, with the range also greater. This can again be attributed to the 20 % gelatine having greater strength.

The 9 mm Luger is not designed to expand on impact; the brass-coated steel full metal jacket stops this from occurring, keeping the projectile intact as it continues through the target. This resulted in complete perforation of the 500 mm target blocks, regardless of the concentration of gelatine. The spin imparted to the individual projectiles designed to keep them stable during flight could be seen to fail during perforation of the gelatine targets, reaching 90° yaw within the 500 mm blocks between two or three times before exiting. This was not a surprising result considering the effect of density on projectile stability [20, 25]. No significant difference in the locations of where 90° yaw occurred for the first and second time corroborates with the fact that no significant difference was found between the locations where maximum temporary cavity expansion occurred and gelatine concentration. This is because the temporary cavity is usually largest when the projectile expands or yaws to 90°; greater projected area causes greater transfer of energy to the tissues [22]. If the projectiles reached 90° yaw a third time, a significant difference in location was found between the two gelatine concentrations. A potential explanation for this is that the denser gelatine produced greater resistance on the projectiles, causing greater deceleration, which in turn led to the projectiles yawing for a third term earlier within the 20 % gelatine blocks.

Instead of an ellipsoid shape, a helical pattern was in 10 % gelatine blocks perforated by 9 mm Luger ammunition. It can be hypothesised that the helical shape was a result of the spin present on the non-deformed projectile, with the larger areas of temporary cavity expansion a result of the projectiles reaching 90° yaw. As a result of the helical shape, only fissure area analysis was carried out on the permanent damage produced. However, this still revealed a similar pattern to that observed with the .223 Remington projectiles; area of damage was significantly greater in 10 % gelatine blocks compared to 20 % gelatine blocks.

The results collected clearly displayed that a difference occurred with regards to the permanent cavity size produced when the same ammunition was tested in different concentrations of gelatine. This result, although not unexpected, does not appear to have been discussed in the open literature before. The permanent cavity left in both concentrations of gelatine was equivalent of the central zone of damage; the area damaged by the direct crushing and lacerating of tissue by both projectile types [18–20]. The calculation of the ellipsoid volume may not be an effective method for deciding the area of living tissue that should be debrided after a gunshot; that should be based on whether tissue is viable or not [21]. However, it was a consistent method for estimating the volume that was damaged and comparing events to see where more damage was done.

### Simulated torso

Porcine samples have been used previously in ballistic testing, in the form of specific sections from whole cadavers (e.g. thigh, abdomen, thorax and neck [27]; thigh [32]; as well as in similar form to the samples tested in this trial [28, 29]). Work conducted by Breeze et al. [32] showed that refrigerating or freezing porcine tissue followed by thawing had no effect on the level of retardation to FSPs. Although work comparing penetration depths of FSPs into 20 % gelatine and porcine tissues has been carried out [27], it is believed that the current work is the first in the open literature to compare damage produced by live rounds in a simulated thorax formed of porcine samples to both 10 and 20 % gelatine.

Comparing .223 Remington baseline shots into porcine tissue and both 10 and 20 % blocks of gelatine revealed significant differences between all three with respect to the distance to the projectile after penetration. However, when shots which failed to strike all sections of the simulant thoracic cavity and/or the ribs were removed, a significant difference was only apparent between the distances to projectiles in 20 % gelatine (in one group) and distances in both 10 % gelatine and the simulated thoracic cavity (both in the same group). The fact that the thoracic cavities had a 10 % gelatine block at the rear of the target and the measurement to the distance of the projectile included the distance travelled through this block is a point of discussion. This target design follows a similar setup used by Fackler et al. [4, 5], however, from which the basis of 10 % gelatine replicating the penetration depth of two projectile types to within 3 % of the penetration depth attained in living porcine leg muscle.

That two shots were removed in order for no significant difference to be present between distance to the projectiles in the simulated thorax, and the 10 % gelatine, was a result of the inhomogeneous nature of tissues which form living organisms. When bone was struck, no significant difference was observed. One of the recommended criteria for a tissue simulant is that it is homogenous, so that factors such as location of shot do not have an effect on the results.

Comment on the temporary cavitation formation in the thorax arrangements was not possible due to the porcine samples being opaque. Therefore, the measure used in this work to compare the two different concentrations of gelatine blocks and the thorax arrangement was depth of penetration, which is a widely accepted measurable criterion used in ammunition lethality studies. However, it should be noted that depth of penetration is not the only criterion considered in lethality studies. Alternatives include energy transfer. Therefore, a limitation of this study is that the energy transfer to tissue (important factor of wounding) could not be directly captured.

The permanent damage produced in the porcine specimens was smaller in scale than that produced in both gelatine blocks. Measurements of entry and exit holes in all porcine samples were the only physical measurements taken; damage in the lungs did not typically extend past the diameters of the penetrating projectiles.

The level of debris collected from the porcine specimens was far greater when compared with the gelatine targets; the presence of solid materials (bone) in the target was the cause of this; not a surprising result. It did, however, demonstrate how the debris can spread when dense materials (such as bone) are present within a target structure that is involved in a gunshot incident. The production of secondary projectiles caused after a bullet striking bone has been reported previously (e.g. [21, 24]). No exterior targets (e.g. clothing, body armour) were struck prior to entering the target, so there was limited chance of foreign debris being brought into the damaged region to cause contamination. However, Hiss and Kahana [24] state that microorganisms from perforated tissues of the target can be spread throughout a wound, causing contamination.

## Conclusions

The damage produced in both concentrations of gelatine was similar in formation for both ammunition types tested, albeit with results on a smaller scaler in 20 % gelatine blocks. This is not a surprising result given the greater density and gel strength of the 20 % blocks. It is of importance, however, given that both concentrations of gelatine are used extensively as tissue simulants of the human body; which is more reminiscent of a human target? Experiments utilising porcine samples to simulate a thorax found depths of penetration to be significantly different to 20 % gelatine, but not 10 % gelatine for expanding rifle ammunition. The level of damage produced in the thoraxes was smaller in scale to the expansion witnessed in both gelatine concentrations, though greater debris was produced in the simulated thoraxes.

## Electronic supplementary material

Below is the link to the electronic supplementary material.

ESM 1 (DOCX 14.0 kb)

## Footnotes

1 Gelita UK Ltd., 3 Macclesfield Road, Cheshire CW4 7NF, UK.

2 Andrews Quality Meats Ltd., 16 High Street, Highworth, Wiltshire, SN6 7AG, UK.

3 Nikon D90, Nikon DX AF-S NIKKOR 18–105 mm lens

4 A2204 Oxford Balance; Analytical products Ltd., Oxford, England, OX3 8ST. Developed, manufactured and tested in compliance with ISO 9001.
