# Supplementary material for: Comparison of porcine thorax to gelatine blocks for wound ballistics studies
Source: Int J Legal Med. 2016 Feb 4;130(5):1353–62. doi: 10.1007/s00414-015-1309-9 (PMC4976058; doi:10.1007/s00414-015-1309-9)
Supplement: Supplementary file 1 [file 414_2015_1309_MOESM1_ESM.docx]

***Details of location and mass of fragments collected from samples after impact by .223 Remington projectiles***

| **Shot number** | **Tract through anterior thoracic wall (g)** | **Front face of lung (g)** | **Tract through lung (g)** | **Back face of lung (g)** | **Tract through posterior thoracic wall (g)** | **Miscellaneous^*^ (g)** |
| --- | --- | --- | --- | --- | --- | --- |
| 1 | 0.0420 |  |  |  | 0.3293, ***0.0030^🌢^*** |  |
| 2 | 1.2417 | 0.1153 |  | 0.0152 | 0.0216 | ***0.0300*** |
| 6 | 0.0981 |  |  |  | ***0.0020*** |  |
| 7 | 0.8323 | 0.2143 |  |  | 0.0467 |  |
| 8 | 0.1025 |  |  | 0.0877 | 0.1055 | 1.5616, ***0.1016*** |
| 9 | 0.6787 |  |  | 0.3732, ***0.0014*** |  |  |
| 10 | 0.0750 | 0.2276, ***0.0252*** | ***0.0249*** | 0.1530 | 0.0380, ***0.0089*** |  |

* Collected from the range immediately after the shot

***^🌢^Fragments in bold and italics were metal fragments***

***Results collected from interactions between 9mm Luger and simulated thoraxes***

| **Shot No.** | **Impact velocity (m/s)** | **Entry location** | | | **Distance through thoracic samples (mm)** | **Total distance (mm)** |
| --- | --- | --- | --- | --- | --- | --- |
|  |  | **Anterior thoracic walls** | **Lungs** | **Posterior thoracic walls** |  |  |
| 1 | 413 | Hit rib 8 | Left lung, bottom area of the inferior lobe | Between ribs 7 & 8 | 177 | > 677 |
| 2 | 420 | Nicked rib 7 | Right lung, bottom area of the inferior lobe | Between ribs 6 & 7 | 177 | > 677 |
| 3 | 410 | Between 7 & 8 | Right lung, middle area of the inferior lobe | Between ribs 5 & 6 | 165 | > 665 |
